# Tongmai Yangxin pill alleviates myocardial no-reflow by activating GPER to regulate HIF-1α signaling and downstream potassium channels

**DOI:** 10.1080/13880209.2023.2184481

**Published:** 2023-03-09

**Authors:** Ting Chen, Yulong Zhang, Manyun Chen, Pu Yang, Yi Wang, Wei Zhang, Weihua Huang, Wei Zhang

**Affiliations:** a Key Laboratory of Hunan Province for Integrated Traditional Chinese and Western Medicine on Prevention and Treatment of Cardio-Cerebral Diseases, Hunan University of Chinese Medicine, Changsha, China; b Department of Clinical Pharmacology, Xiangya Hospital, Central South University, Changsha, People's Republic of China; c Hunan Key Laboratory of Pharmacogenetics, Institute of Clinical Pharmacology, Central South University, Changsha, People's Republic of China; d Engineering Research Center of Applied Technology of Pharmacogenomics, Ministry of Education, Changsha, People's Republic of China; e Institute of Traditional Chinese medicine, Tianjin University of Traditional Chinese medicine, Tianjin, People's Republic of China; f Department of General Surgery, Xiangya Hospital, Central South University, Changsha, People's Republic of China

**Keywords:** Cardioprotective effects, molecular mechanisms, network pharmacology, coronary microvasculature

## Abstract

**Context:**

The Tongmai Yangxin pill (TMYX) has potential clinical effects on no-reflow (NR); however, the effective substances and mechanisms remain unclear.

**Objective:**

This study evaluates the cardioprotective effects and molecular mechanisms of TMYX against NR.

**Materials and methods:**

We used a myocardial NR rat model to confirm the effect and mechanism of action of TMYX in alleviating NR. Sprague-Dawley (SD) rats were divided into Control (Con), sham, NR, TMYX (4.0 g/kg), and sodium nitroprusside (SNP, 5.0 mg/kg), and received their treatments once a day for one week. *In vitro* studies in isolated coronary microvasculature of NR rats and *in silico* network pharmacology analyses were performed to reveal the underlying mechanisms of TMYX and determine the main components, targets, and pathways of TMYX, respectively.

**Results:**

TMYX (4.0 g/kg) showed therapeutic effects on NR by improving the cardiac structure and function, reducing NR, ischemic areas, and cardiomyocyte injury, and decreasing the expression of cardiac troponin I (cTnI). Moreover, the mechanism of TMYX predicted by network pharmacology is related to the HIF-1, NF-κB, and TNF signaling pathways. *In vivo*, TMYX decreased the expression of MPO, NF-κB, and TNF-α and increased the expression of GPER, p-ERK, and HIF-1α. *In vitro*, TMYX enhanced the diastolic function of coronary microvascular cells; however, this effect was inhibited by G-15, H-89, L-NAME, ODQ and four K^+^ channel inhibitors.

**Conclusions:**

TMYX exerts its pharmacological effects in the treatment of NR *via* multiple targets. However, the contribution of each pathway was not detected, and the mechanisms should be further investigated.

## Introduction

Primary percutaneous coronary intervention (PCI) is the preferred treatment for acute myocardial infarction (AMI) (Li et al. 2021). However, a sizable proportion of PCI patients achieve epicardial coronary artery reperfusion but not myocardial reperfusion, a condition known as no-reflow (NR) (Annibali et al. 2022). Poor or non-existent perfusion of myocardial tissue can lead to malignant arrhythmia, heart failure, and life-threatening events in patients. Unfortunately, the incidence of NR in patients with AMI after PCI is as high as 30% (Niccoli et al. 2009; Xenogiannis et al. 2021). Therefore, it is necessary to identify novel therapeutic agents and targets.

The Tongmai Yangxin pill (TMYX), a traditional Chinese patent medicine, which is composed of the classic formulas ‘Zhigancao Decoction’ and ‘Shengmaiyin’, could be used for NR (Chen et al. 2020, 2021). The medication consists of the following 11 herbs: *Rehmannia glutinosa* (Gaert.) Libosch. ex Fisch. et Mey. (Scrophulariaceae) (Dihuang)*, Spatholobus suberectus* Dunn (Leguminosae) (Jixueteng)*, Pleuropterus multiflorus* (Thunb.) Nakai (Polygonaceae) (Zhiheshouwu)*, Equus asinm* L. (Equidae) (Ejiao)*, Ophiopogon japonicus* (L. f.) Ker-Gawl. (Liliaceae) (Maidong)*, Codonopsis pilosula* (Franch.) Nannf. (Labiatae) (Dangshen)*, Chinemys reevesii* (Gray) (Batagurinae) (Cuguijia)*, Cinnamomum cassia* Presl (Lauraceae) (Guizhi)*, Ziziphus jujuba* Mill. (Rhamnaceae) (Dazao)*, Schisandra chinensis* (Turcz.) Baill. (Dicotyledoneae) (Wuweizi), and *Glycyrrhiza uralensis* Fisch. (Leguminosae) (Gancao). The active ingredients of these drugs include flavonoids, saponins, and lignans (Fan et al. 2016). Clinical trials have shown that TMYX can effectively improve the clinical symptoms of patients with coronary heart disease and atrial arrhythmia, and prevent the development of cardiac remodeling and dysfunction (Wang et al. 2020). The anti-inflammatory activity of TMYX in the treatment of coronary heart disease is associated with the estrogen receptor and NF-κB signaling pathways (Fan et al. 2021). TMYX exerts therapeutic effects on stable angina by improving myocardial energy supply disorder and amino acid dysfunction and attenuating oxidative stress and inflammation (Cai et al. 2018). Moreover, preliminary research has confirmed that TMYX could significantly reduce myocardial ischemia-reperfusion with NR, which is related to myocardial enzyme activity in serum, oxidative stress, post-cardiac load, and myocardial pathological damage (Chen et al. 2020, 2021). Therefore, TMYX has a potential therapeutic effect on NR. However, investigation of the protective mechanism of NR by TMYX is still difficult due to its complex components, multiple targets, and pathways.

A network pharmacology method is a promising approach for systems biology-based technologies and multiple omics (Kibble et al. 2015; Li et al. 2021). In this study, network pharmacology with experimental validation was used to clarify the underlying mechanisms of TMYX in NR after myocardial ischemia-reperfusion. First, we used an NR rat model after myocardial ischemia-reperfusion to validate the cardioprotective effects of TMYX (4.0 g/kg) (Wang et al. 2011). We then constructed an herb-compound-target interaction network to estimate the active ingredients, potential targets, and pathways of TMYX. Finally, we used an NR after myocardial ischemia-reperfusion rat model and isolated the coronary microvasculature to validate the mechanisms of TMYX in alleviating NR.

## Materials and methods

### Animals and myocardial NR model

Male Sprague-Dawley rats (250 ± 10 g) were obtained from Beijing Weitonglihua Experimental Animal Technology Co., Ltd., (certificate number: 11401300051612; SCXK20160006, China). All experimental protocols were conducted in accordance with the guidelines approved by the Animal Care Committee of the Tianjin University of Traditional Chinese Medicine (TCM-LAEC2019095). After 7 d of adaptive feeding, a myocardial NR model was established. After anesthesia, the left anterior descending coronary artery (LAD) was found between the 3rd and 4th costal cartilages of the left margin of the sternum and ligated with a 5/0 suture 2–3 mm below the left atrial appendage. The sham-operated group underwent surgery without ligation. The heart was quickly returned to the chest cavity and the exhaust was vented. After ligation for 2 h, the ligation line was released for reperfusion. Echocardiography was performed 2 h after reperfusion; the success of the model was marked by an ejection fraction (EF) value of < 50% and obvious myocardial NR (Hernández-Reséndiz et al. 2015; Quan et al. 2021).

### Grouping and drug administration

After reperfusion for 2 h, the rats were randomly assigned according to the EF value (<50%). The rats were assigned to five treatment groups: Con, sham, NR, TMYX (4.0 g/kg), and SNP (5.0 mg/kg, positive control group) (*n* = 16). The rats in the Con, Sham, and NR groups were administered the same volume of 0.5% carboxyl methyl cellulose (CMC-Na) (Batch No. C8621, Beijing Solarbio Technology Co., Ltd., China) solvent, the rats in the SNP group were injected intraperitoneally, and the rats in the TMYX groups received their treatments intragastrically for one week. The first administration time was 4 h after modeling. The volume of administration was 1 mL/100 g.

### Drug preparation and determination of components in TMYX

TMYX (Batch No. 1070353) was provided by the Tianjin Zhongxin Pharmaceutical Group Co., Ltd., Lerentang Pharmaceutical Factory (Tianjin, China). The prescription and preparation of TMYX were consistent with those in the Chinese Pharmacopoeia (2020 Edition). Briefly, *Rehmanniae Radix, Ophiopogonis Radix, Glycyrrhizae Radix et Rhizoma, Polygoni Multiflori Radix Praeparata, Asini Corii Colla*, and *Cinnamomi Ramulus* were crushed into fine powders. The other five ingredients, *Spatholobi Caulis, Schisandrae chinensis Fructus, Codonopsis Radix, Testudinis Carapax et Plastrum*, and *Jujubae Fructus*, were boiled twice in distilled water for 3 h each. After filtering, the filtrate was condensed into a thick paste, added to the prepared fine powder, stirred well, and dried to make 450 g pills. After the pills were made, 25.6 g TMYX was weighed and soaked in 64 mL of ultrapure water containing 0.5% CMC-Na (Batch No. C8621; Beijing Solarbio Technology Co., Ltd., Beijing, China). After softening, TMYX was fully dissolved by shocking, and a 0.4 g/mL solution was prepared for oral administration. SNP (Batch No. 71778-25 G) was purchased from Sigma-Aldrich (St. Louis, MO, USA). UPLC-Q-TOF-MS/MS was used to analyze the chemical constituents of TMYX. The methods, machine equipment, and standard solutions have been previously described (Chen et al. 2020). A total of 32 compounds were identified according to the molecular fragment peaks, control retention time, and secondary spectra (Chen et al. 2021).

### Measurement of the area of NR and ischemia

A 1 mL/kg volume of 6% thioflavin S (Batch No. T1892-25G, Sigma, USA) was injected into the rat inferior vena cava. One minute after the thioflavin S injection, the LAD was ligated *in situ* and 2% Evans Blue (Batch No. E2129-10G, Sigma, USA) at 1 mL/kg was injected into the inferior vena cava (Li et al. 2022). The heart was removed and frozen at −20 °C for 10 min. The heart was evenly divided into five pieces. Myocardial slices were then incubated in a 37 °C incubator with 1% TTC (Batch No. T8170, Solarbio, China) solution for 30 min. The fluorescent region was then observed under a light source with a wavelength of 365 nm. The fluorescent area is the reflow area and the non-fluorescent area is the NR area. Under ordinary light, the blue-stained area is a non-ischemic area and the non-blue-stained area is an ischemic area. The light red area represents the ischemic, non-infarcted myocardial area, and the gray-white area represents the infarcted myocardial area. Each area was measured using Image-Pro Plus 6.0.

### Measurement of cardiac structure and function in rats

The function and structure of the rat heart were measured using echocardiography (Vevo 2100, VisualSonics, Canada). The probe frequency was set to 12 Mhz. After anesthesia, the rats were fixed on a rat plate, and the chest was prepared. The probe was coated with a coupling agent (YY 0299, Tianjin, China) for examination and placed on the left side of the sternum, forming an angle of 30 with the sternum midline. The M-type sample line was perpendicular to the interventricular septum and posterior wall of the left ventricle to obtain M-type echocardiography (Chen et al. 2021). Measurements included the left ventricular ejection fraction (EF), left ventricular shortening rate (FS), left ventricular mass (LV mass), left ventricular end-diastolic volume (LV Vold), left ventricular end-systolic volume (LV Vols), left ventricular peak flow velocity (LVOT peak), and left ventricular stroke volume (LVSV). All measured values were averaged over the three cardiac cycles.

### Detection of cardiac troponin I (cTnI) activity in the serum of rats with NR

After 1 h of treatment administration on the third day, blood was collected from the inner canthus of each rat in each group. After 30 min, the serum was centrifuged (Thermo Scientific, Waltham, MA, USA) at 3500 rpm, 4 °C for 10 min. The serum was analyzed using a rat cardiac troponin I (TNNI3) detection kit (Batch No. SEA478Ra, Uscnk, China) to evaluate cTnI, a heart muscle damage indicator (Bai et al. 2021).

### Detection of the histopathological changes in myocardial tissue in NR rats by HE staining

The rats were euthanized, and their hearts were removed. Each heart was embedded in paraffin. Sections of 4 μm thickness were cut. After dewaxing and dehydration, myocardial sections were stained with hematoxylin and eosin and observed under a microscope.

### Constructing a database of material basis

All constituents of TMYX were obtained from the Traditional Chinese Medicine Systems Pharmacology Database and Analysis Platform (http://lsp.nwu.edu.cn/tcmsp. php, TCMSP) and Batman-TCM (http://bionet.ncpsb.org.cn/batman-tcm/) (Chen et al. 2021). Oral bioavailability (OB) and drug-likeness (DL) indices recommended by the TCMSP were employed to verify the drug ability of each candidate (Su et al. 2022). High OB appears to be more likely to be a drug-like ingredient. The DL index was used to assess the chemical suitability of the compounds (Hu et al. 2021). As TCMSP suggests, molecules with OB ≥ 30% and DL ≥ 0.18 were preserved as bioactive compounds.

### Mining NR after myocardial ischemia-reperfusion associated targets and target genes

Protein targets associated with NR after myocardial ischemia-reperfusion were provided by the GeneCard, OMIM, PharmGkb, TTD databases with ‘NR after myocardial ischemia-reperfusion’ as the keywords. All the targets were only limited to ‘*Homo sapiens*’. Subsequently, the protein names of all targets were switched to the corresponding gene names on the UniProt website (http://www. uniprot.org). The same procedure was performed to extract the relevant target genes (drug targets) of TMYX herbs. Then, the overlapping genes (network target) (Li et al. 2021) of the relevant target genes of TMYX and NR after myocardial ischemia-reperfusion-associated target genes were screened using a vein diagram.

### Conducting protein–protein interaction (PPI) network

To elucidate the interaction of the therapeutic target genes and identify the hub genes, we imported the therapeutic genes into the Search Tool for the Retrieval of Interacting Genes/Proteins database (STRING, version 11.0, https://string-db.org/, version:11.0), selected ‘*Homo sapiens*’ as the species and set the parameter at the highest confidence (0.900) level to obtain PPI data. The hub genes were identified using topology analysis. PPI network visualization and topology analysis were performed using Cytoscape software (Hou et al. 2022).

### Kyoto encyclopedia of genes and genomes (KEGG) enrichment analysis

The R 4.0.0 was used for KEGG enrichment analysis. The R package including ‘colorspace,’ ‘stringi,’ ‘ggplot2’ and a bioconductor package that includes ‘DOSE,’ ‘clusterProfiler,’ ‘enrichplot’ were installed in software R 4.0.0. The database was org. Hs.eg.db (DOI:10.18129/http://b9.bioc.org.Hs.eg.db); the ‘enrich-KEGG’ function was applied for KEGG enrichment analysis, and the database was the KEGG database (https://www.kegg.jp/). As for the parameters of the two functions, species was set to ‘has,’ and the filter values (i.e. *p* value and *q*-value) were set to 0.05 (Wei et al. 2020). The first 20 enrichment results were visualized as a bar graph and the KEGG regulatory network was generated using Cytoscape 3.8.0 software.

### Validation of compound‐target interaction

The three-dimensional (3D) structure of the target protein was downloaded from the PDB database (https://www.rcsb.org/). The target protein was processed by removing ligand and water motifs and adding hydrogen using AutoDockTools-1.5.6. The three-dimensional structures of the candidate compounds were downloaded from PubChem (https://pubchem.ncbi.nlm.nih.gov/). The protein receptors and ligands were converted to the PDBQT format using AutoDockTools. Finally, AutoDock Vina software was used for molecular docking and the lowest free energy model was selected for visual analysis using PyMOL and Discovery Studio 2016 Client. A docking score < −5 indicated good binding affinity (Yan et al. 2017).

### Detection of the diastolic function of the isolated coronary microvascular in NR rats

The microvascular ring of the left anterior descending branch was suspended between two parallel steel hooks in an organ bath. To keep the blood vessels alive, the organ bath was maintained at 37.0 °C and bubbled with 95% O_2_ and 5% CO_2_. After vascular balance, 5 mL of the KPSS solution (composition and batch numbers listed in Table 1) was used to stimulate the blood vessels to reach the vascular ring leveling stage, and the PSS buffer (composition and batch numbers listed in Table 2) was used to wash the blood vessels 2 times/10 min. KPSS stimulation of the blood vessels was repeated twice, and if the contractile tension was >2 mN, the vascular ring was considered to exhibit good activity. Vascular tension was recorded using a microvascular tension sensor (Danish Myo Technology A/S). In order to observe whether TMYX can alleviate NR by activating GPER to regulate the HIF-1α pathway and downstream potassium channel, a GPER blocker (G-15, Batch No. 1161002-05-6, MCE, China), PKA blocker (H-89, Batch No. B 1427, Sigma, USA), eNOS blocker (L-NAME, Batch No. N 5751, Sigma, USA), sGC inhibitor (ODQ, Batch No. O 3636, Sigma, USA), and four K^+^ channel (calcium-activated potassium channel, ATP-sensitive potassium channel, inward rectifier potassium channel, and voltage-dependent potassium channel) inhibitors (TEA: Batch No. T2265-25G, Gli: Batch No. G0639-5G-9, Bacl_2_: Batch No. 202738-5 G, 4-AP: Batch No. 275875-1 G, Sigma, USA) were administered to the isolated coronary microvasculature.

**Table 1.** Composition and batch numbers of KPSS solution.

Composition	Weight(g)	batch numbers
Sodium chloride	2.18	T 1226 (Tianjin Zhiyuan Chemical Reagent Co., Ltd., China)
Potassium chloride	2.24	T 646 (Tianjin Guangfu Technology Development Co., Ltd., China)
Potassium dihydrogen phosphate	0.08	T 1274 (Tianjin Guangfu Technology Development Co., Ltd., China)
MgSO_4_7H_2_O	0.145	10034-99-8 (Beijing sulaibao Technology Co., Ltd., China)
Sodium bicarbonate	0.625	T 640 (Tianjin Guangfu Technology Development Co., Ltd., China)
Glucose water	0.5	T 3475 (Tianjin Guangfu Technology Development Co., Ltd., China)
EDTA	0.005	60-00-4 (Beijing sulaibao Technology Co., Ltd., China)
Crystalline calcium chloride	5.55	T 19001 (Tianjin Guangfu Technology Development Co., Ltd., China)

**Table 2.** Composition and batch numbers of PSS buffer.

Composition	Weight(g)	batch numbers
Sodium chloride	7.598	T 1226 (Tianjin Zhiyuan Chemical Reagent Co., Ltd., China)
Potassium chloride	0.35	T 646 (Tianjin Guangfu Technology Development Co., Ltd., China)
Potassium dihydrogen phosphate	0.16	T 1274 (Tianjin Guangfu Technology Development Co., Ltd., China)
MgSO_4_7H_2_O	0.29	10034-99-8 (Beijing sulaibao Technology Co., Ltd., China)
Sodium bicarbonate	1.25	T 640 (Tianjin Guangfu Technology Development Co., Ltd., China)
Glucose water	1.00	T 3475 (Tianjin Guangfu Technology Development Co., Ltd., China)
EDTA	0.01	60-00-4 (Beijing sulaibao Technology Co., Ltd., China)
Crystalline calcium chloride	5.55	T 19001 (Tianjin Guangfu Technology Development Co., Ltd., China)

### Immunohistochemistry of MPO

Tissue sections (4 μm thick) of cardiac tissue were mounted on polylysine-coated slides. The paraffin sections were dewaxed using a standard method and incubated for 10 min with 3% hydrogen peroxide (H_2_O_2_). Each section was incubated with blocking serum (Batch No. AR0004, Boster Biological Technology Co., Ltd., China) at room temperature for 30 min, followed by incubation with a primary myeloperoxidase antibody (1:200 dilution, Batch No. Bs-4943R; Beijing Biosynthesis Biotechnology Co., Ltd., China) overnight at 4 °C. Sections incubated in phosphate-buffered saline (PBS) without antibodies were used as negative controls. After incubation with biotinylated secondary antibodies, the sections were incubated with an avidin-biotin complex reagent containing horseradish peroxidase for 30 min. The sections were then stained with 3,3′-diaminobenzidine (DAB) (Batch No. AR1022; Boster Biological Technology Co. Ltd., China). The Image-Pro Plus 6.0 System image analysis system was used for quantitative analysis (Kin et al. 2004).

### Real-time PCR

Total RNA was extracted from rat myocardial tissues using TRIzol (1 mL) (Batch No. 135406, Life Technologies, USA). RNA was reverse-transcribed according to the instructions of the GoScript Reverse Transcription System Kit (Batch No. A5001, Promega, USA). Quantitative PCR was performed using 2× SYBR Green Qpcr Master Mix (Batch No. B21203, Bimake, USA). Briefly, complementary DNA amplification conditions were as follows: initial activation at 95 °C for 10 min, 95 °C for 15 s, 60 °C for 1 min for 40 cycles, and 95 °C for 15 s, 60 °C for 15 s, and 95 °C for 15 s. The results were analyzed using the 2^−ΔΔCT^ method, with GAPDH as the internal control. Primer sequences used in this study are listed in Table 3.

**Table 3.** The real-time RT-PCR oligonucleotide primers.

Gene	Primer	Sequence (5′–3′)
GAPDH	Forward	ACGGCAAGTTCAACGGCACAG
	Reverse	CGACATACTCAGCACCAGCATCAC
GPER	Forward	TCAGCAGTACGTGATTGCCCTCTT
	Reverse	ACTGCTCTGTGCTGTCTGGAATGA
HIF-1α	Forward	TCACAAATCAGCACCAAGCAC
	Reverse	AAGGGGAAAGAACAAAACACG
ERK	Forward	TACCGAGCCCCAGAGATCAT
	Reverse	GGAAGATAGGCCGGTTGGAG
NF-κB	Forward	GCTTTGCAAACCTGG GAATA
	Reverse	CAAGGTCAGAAT GCACCAGA
TNF-α	Forward	CAGGCGGTGCCTATGTCTC
	Reverse	CGATCACCCCGAAGTTCAGTAG

### Western blot analysis

The western blot protocol and semiquantitative analysis were performed as previously described (Chen et al. 2021). The antibodies used were rabbit monoclonal GPER (1:1000 dilution; Batch No. bs-1380R, Bioss, China), rabbit polyclonal HIF-1α (1:1000 dilution, Batch No. 340462, Hunan Kehang Biotech Inc., China), rabbit monoclonal ERK (1:1000 dilution, Batch No. 9102S, CST, USA), rabbit monoclonal p-ERK (1:1000 dilution, Batch No. 9106S, CST, USA), rabbit monoclonal NF-κB (1:1000 dilution, Batch No. bs-34045R, Beijing Biosynthesis Biotechnology Co., Ltd., China), rabbit monoclonal TNF-α (1:1000 dilution, Batch No. ab205587, Abcam, USA), rabbit monoclonal GAPDH (1:1000 dilution, Batch No. 5174 T, CST, USA), and anti-rabbit IgG (1:5000 dilution, Batch No. 7074P2, CST).

### Statistical analysis

Statistical analyses were performed using SPSS version 21.0. All data are expressed as the mean standard deviation and were analyzed by one-way analysis of variance (ANOVA) followed by the least significant difference (LSD) or Dunnett’s T3 test. Differences were considered statistically significant when the *p*-value was less than 0.05.

## Results

### Effects of TMYX on the NR myocardial area and ischemic myocardial area in NR rats

**Figure 1.**
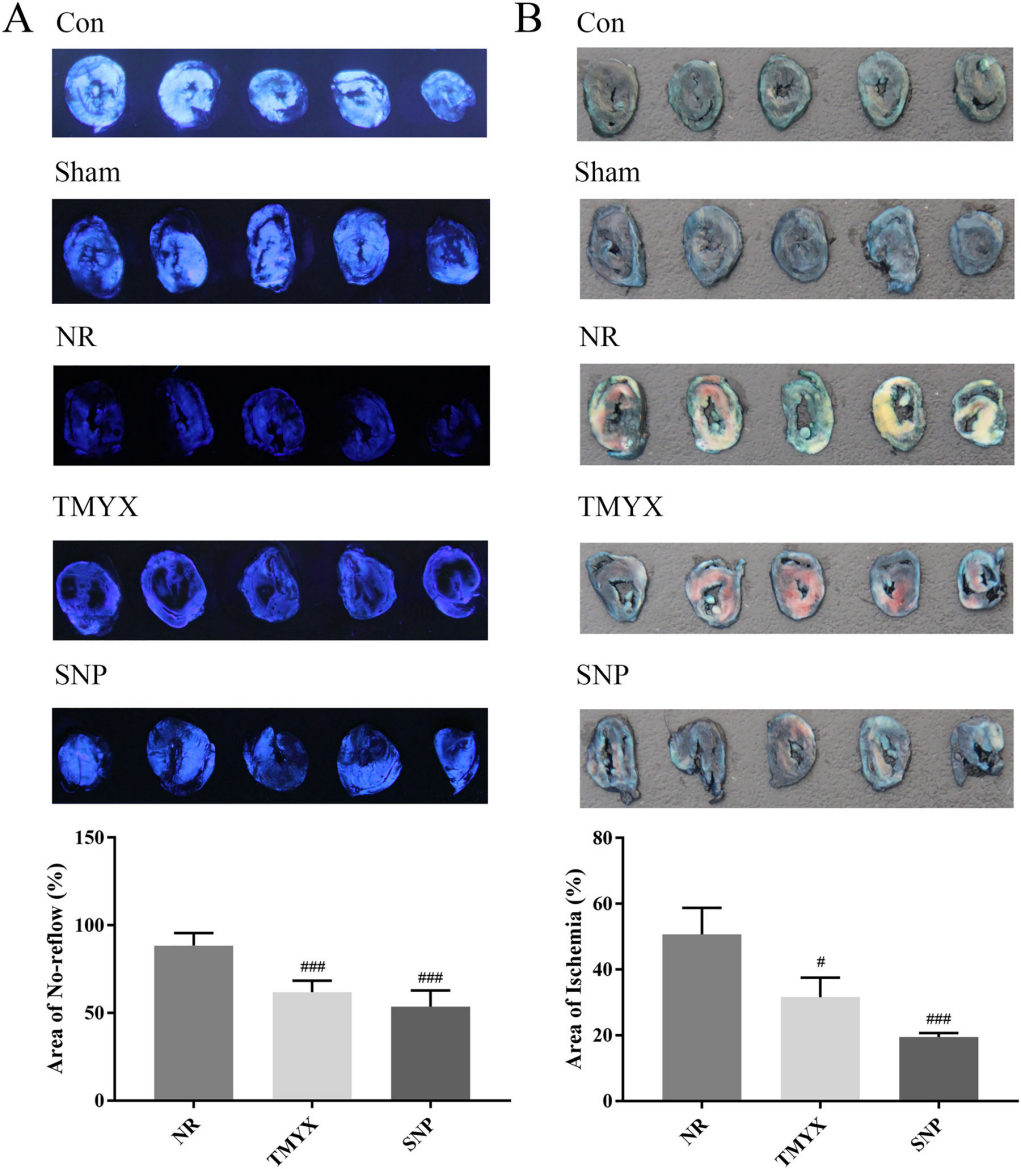
Effects of TMYX on NR myocardial area and ischemic myocardial area in NR rats. (A) Effects of TMYX on NR myocardial area in NR rats. (B) Effects of TMYX on the ischemic myocardial area in NR rats. The data are expressed as the mean ± SD; *n* = 5 animals/group; ^#^*p* < 0.05, ^###^*p* < 0.001 vs. NR group.

To study the *in vivo* role of TMYX alleviating NR, we first used thioflavin S, Evans Blue and TTC staining to observe NR myocardium and ischemic myocardium area. The results showed that the areas of the NR myocardium and ischemic myocardium in the NR group were 88.4% and 50.7%, respectively (Figure 1(A–B)). Compared with those in the NR group, the area of the NR myocardium and ischemic myocardium decreased to 61.8% and 31.6%, respectively, in the TMYX group (*p* < 0.05, *p* < 0.001), and the area of the NR myocardium and ischemic myocardium decreased to 53.6% and 19.5%, respectively, in the SNP group (*p* < 0.001). The pharmacodynamics data indicate the efficacy of TMYX alleviating NR *in vivo*.

### Effect of TMYX on the cardiac structure and function of NR rats

**Figure 2.**
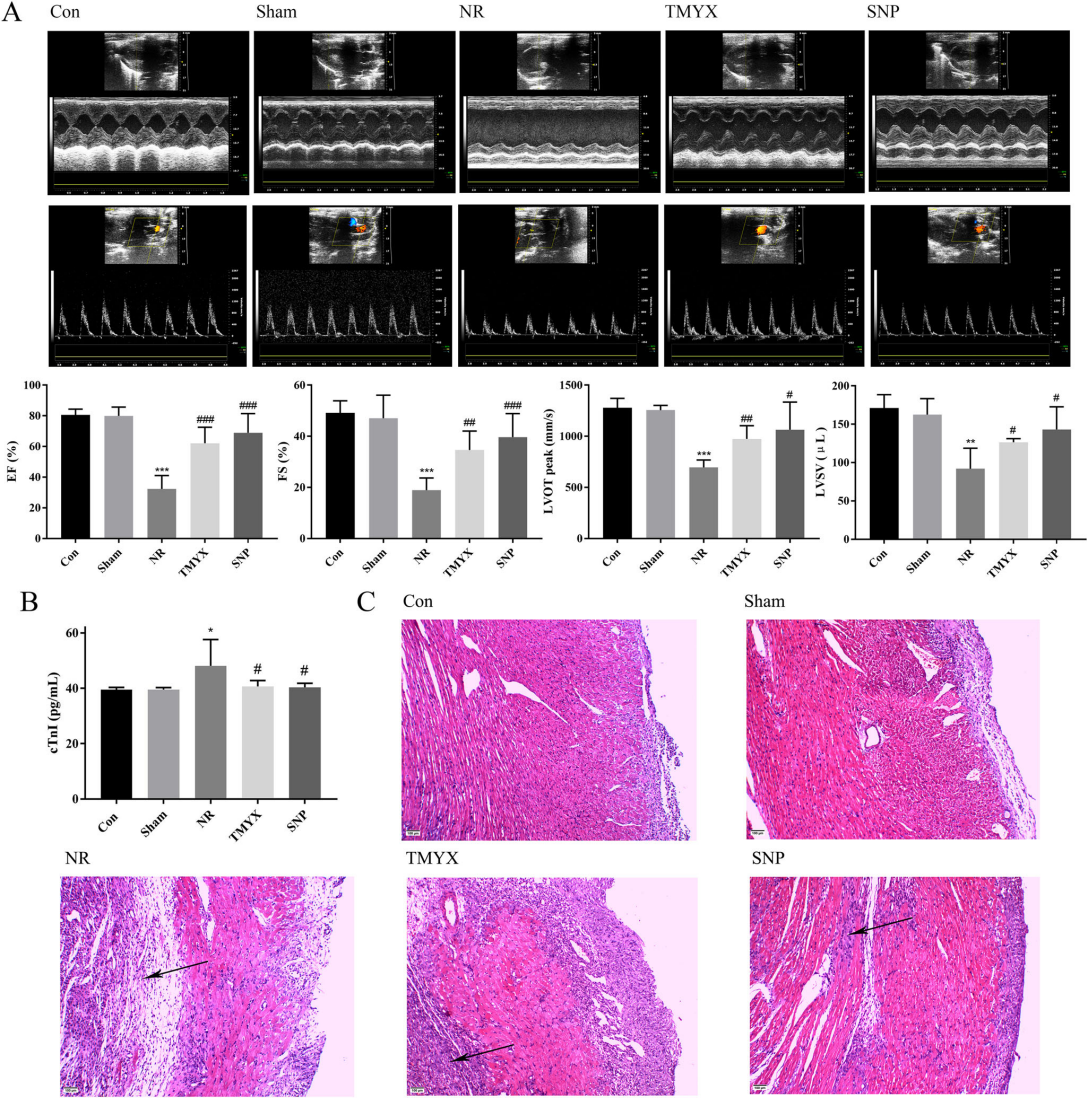
Effect of TMYX on NR rats. (A) Effect of TMYX on the cardiac structure and function of NR rats. (B) Effect of TMYX on myocardial enzyme activity in NR rats. (C) Effect of TMYX on the pathological changes in NR rats (×100, *n* = 3). The data are expressed as the mean ± SD; *n* = 16 animals/group; *p < 0.05, **p < 0.01, ***p < 0.001 vs. Sham group; ^#^*p* < 0.05, ^##^*p* < 0.01, ^###^*p* < 0.001 vs. NR group.

To investigate whether TMYX improved cardiac structure and function *in vivo*, we next measured the function and structure of the rat heart using echocardiography. As shown in Figure 2(A), compared with those in the Con group, the sham operation had no effect on cardiac structure and function. Compared to those in the Sham group, the EF, FS, LVOT peak, and LVSV in the NR group were significantly decreased (*p* < 0.01, *p* < 0.001). However, the EF, FS, LVOT peak, and LVSV in the TMYX and SNP groups were significantly higher than those in the NR group (*p* < 0.05, *p* < 0.01, and *p* < 0.001). These results demonstrated that TMYX could improve cardiac structure and function in NR rats.

### Effect of TMYX on myocardial enzyme activity in NR rats

In AMI, cTnI is released as a result of myocardial cell necrosis and can be used as an indicator for evaluating myocardial injury. As shown in Figure 2(B), compared with those in the Con group, the sham operation had no effect on cTnI activity. However, cTnI activity in the NR group was significantly higher than that in the Sham group (*p* < 0.05). Compared with the NR group, the activity of cTnI in the TMYX and SNP groups was significantly decreased (*p* < 0.05). These data indicated TMYX could alleviate myocardial injury and protect myocardial tissue.

### Effect of TMYX on the pathological changes in the myocardial tissues in NR rats

To further observe the effect of TMYX on the pathological changes in the myocardial tissues, we performed HE staining. The Con and Sham groups exhibited complete myocardial cells, and the myocardial fibers were arranged neatly, tightly, and crisscrossed with each other (Figure 2(C)). The NR group showed disordered myocardial cells, dissolved nuclear pyknosis, muscle fiber swelling, and extensive inflammatory cell infiltration. The degree of myocardial injury in the TMYX and SNP groups was significantly reduced, the area of the lesion was obviously reduced, oedema was significantly reduced, infiltration of a small number of inflammatory cells and interstitial oedema was observed, and vacuolated cells were occasionally observed.

### Ingredient targets–disease targets network analysis

**Figure 3.**
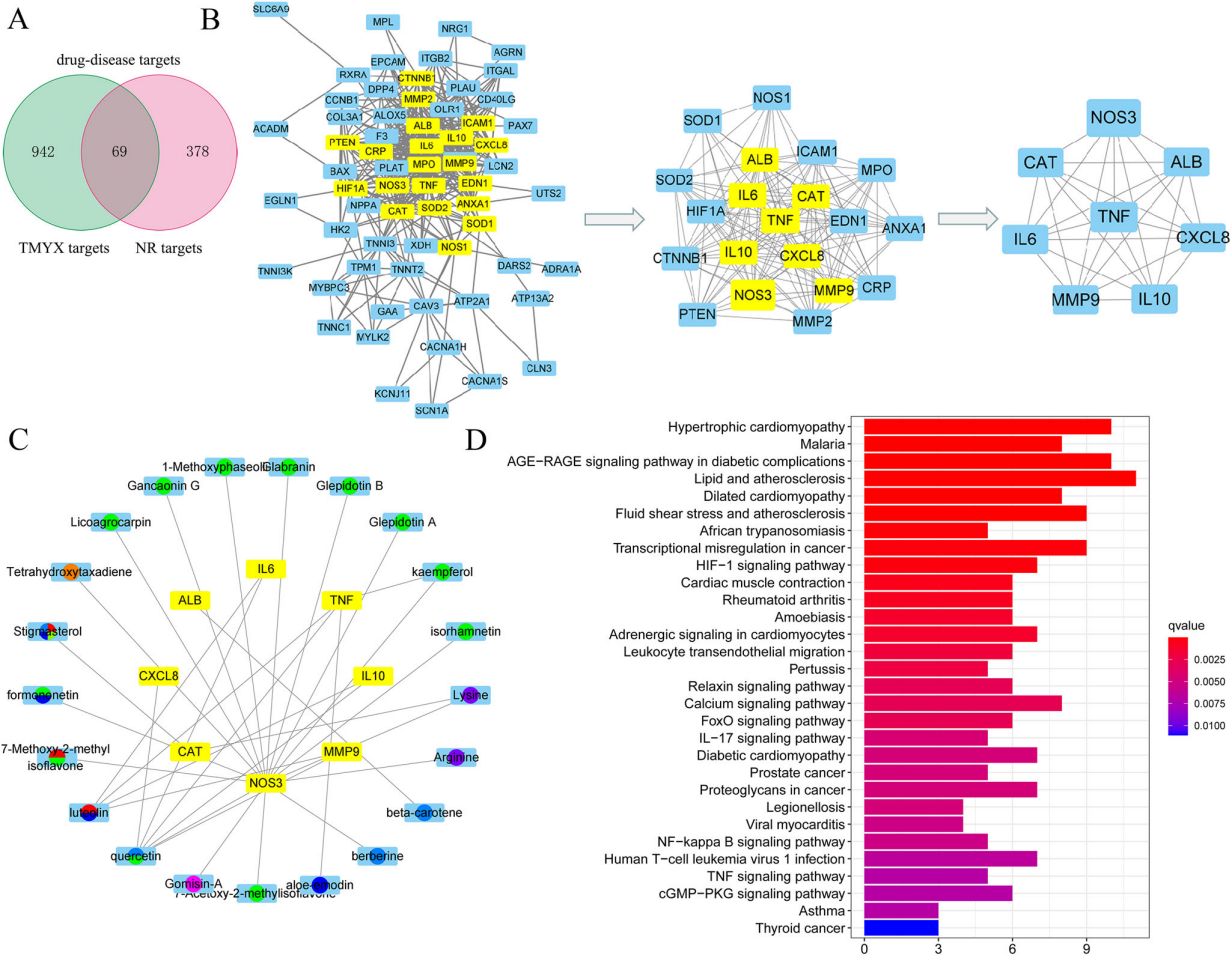
The ingredient–target network and KEGG enrichment analysis of TMYX on NR. (A) The Venn diagram of drug targets and NR-related targets. (B) PPI network core analysis. (C) The active compounds of TMYX and major targets. The yellow rectangles in the middle represent drug-disease targets; the green, cyan, orange, blue, yellow-green, red, purple, wathet blue, yellow, dark purple represent the active ingredients from *Glycyrrhizae Radix et Rhizoma, Cinnamomi Ramulus, Polygoni Multiflori Radix Praeparata, Spatholobi Caulis, Ophiopogonis Radix, Codonopsis Radix, Schisandrae chinensis Fructus, Jujubae Fructus, Rehmanniae Radix,* and *Asini Corii Colla*. (D) KEGG enrichment analysis of TMYX targets in alleviating NR. The horizontal axis of the KEGG diagram represents the gene proportion enriched in each entry, and the color shows the enrichment degree according to the corrected P value.

In total, 447 NR targets after myocardial ischemia-reperfusion were obtained from the GeneCard, OMIM, PharmGkb, and TTD databases. Moreover, 69 overlapping genes of potential targets of TMYX and NR-related targets were regarded as drug-disease targets (Figure 3(A)). Then we conducted a PPI network to elucidate the interaction of the therapeutic target genes and identify the hub genes. In the PPI network, the degree centrality (DC) and betweenness centrality (BC) of target proteins were calculated using topological analysis. The DC and BC reflect the influence of the corresponding node in the entire network. The combination of DC and BC values has been confirmed to be effective for screening reliable proteins (Kibble et al. 2015). As shown in Figure 3(B), 65 protein nodes and 389 edges were obtained for the intersection genes. After screening with DC and BC (gene conditions greater than the median), the first 20 proteins are shown in Table 4 (in descending order of degree), with 152 edges. Among the 65 proteins, eight were predicted targets of the active ingredients, with their corresponding genes being *NOS3*, *ALB*, *CXCL8*, *IL10*, *MMP9*, *IL6*, *CAT*, and *TNF*. The different active components corresponded to the eight proteins (Figure 3(C)), which reflected the characteristics of multi-component, multi-target TMYX. Among them, the degree values of quercetin, luteolin, kaempferol, and lysine were 6, 4, 2 and 2, respectively, indicating that they were the most important active components in the network (Table 5).

**Table 4.** Topological analysis results by degree—the first 20 proteins.

Gene names	Annotation	Degree	Betweenness
ALB	Protein albatross	19	9.274314574
CAT	Catalase	19	9.274314574
IL6	Interleukin-6	19	9.274314574
TNF	Tumor necrosis factor	19	9.274314574
IL10	Interleukin-10	18	6.413997114
NOS3	Nitric oxide synthase, endothelial	18	7.210678211
CXCL8	Interleukin-8	17	3.561616162
MMP9	Matrix metalloproteinase-9	17	3.561616162
ICAM1	Intercellular adhesion molecule 1	16	2.518686869
EDN1	Endothelin-1	16	4.065512266
HIF1A	Hypoxia-inducible factor 1-alpha	16	4.261183261
MMP2	Matrix metalloproteinase-2	16	2.533838384
SOD2	Superoxide dismutase 2	14	2.924603175
CTNNB1	Catenin beta-1	14	0.681818182
MPO	Myeloperoxidase	13	0.363636364
PTEN	Phosphatase and tensin homolog	12	0.166666667
CRP	C-reactive protein	12	0
ANXA1	Annexin A1	11	0
SOD1	Superoxide dismutase 1	9	0.222222222
NOS1	Nitric oxide synthase, brain	9	0.416666667

**Table 5.** Degree value and virtual docking of 18 vital active compounds from TMYX for no-reflow targets.

Compound	Degree	Binding affinity/ (kcal/mol)	
		NOS3	TNF
7-Methoxy-2-methyl isoflavone	1	−9.0	–
Berberine	1	−10	–
Quercetin	6	−9.1	−7.7
Isorhamnetin	1	−9.3	–
Formononetin	1	−8.4	–
Kaempferol	2	−9.5	−7.8
Glepidotin A	1	−10	–
Glepidotin B	1	−9.8	–
Glabranin	1	−10.3	–
1-Methoxyphaseollidin	1	−9.2	–
7-Acetoxy-2-methylisoflavone	1	−9.5	–
Gancaonin G	1	−9.8	–
Licoagrocarpin	1	−9.1	–
Gomisin-A	1	−8.5	–
Arginine	1	−6.2	–
Lysine	2	−5.3	–
Luteolin	4	–	−8.1
Aloe-emodin	1	–	−7.9

### Analysis of KEGG enrichment of related targets and verification of compound-target interaction

**Figure 4.**
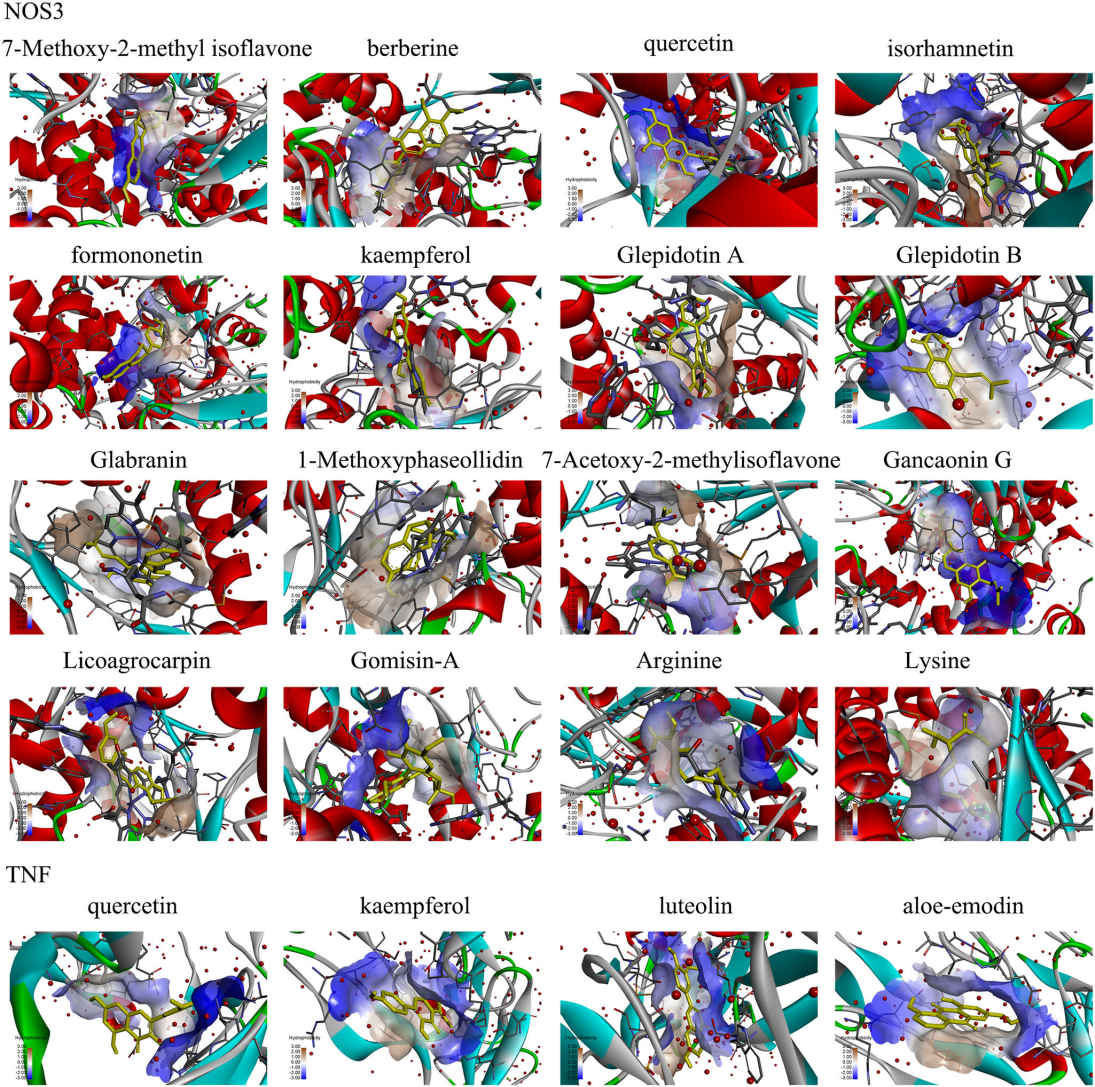
The conformations of main active compounds and major hub targets.

KEGG pathway enrichment analysis was conducted using the R package. The target pathway was built to delve into the mechanisms of potential targets acting on their corresponding signaling pathways. As suggested by these results (Figure 3(D)), TMYX probably exerted therapeutic effects on NR by regulating signaling pathways, including the HIF-1, cGMP-PKG, NF-kappa B, and TNF signaling pathways. Then, we used the molecular docking method to predict the binding affinity of active compounds and major protein targets. The binding affinity results are presented in Table 5. The conformations of the key active compounds and major hub targets are shown in Figure 4. The conformations of active compounds and major protein targets (NOS3 and TNF) showed good binding interactions, which were also reliable.

### Effect of TMYX on the expression of the GPER, ERK, HIF-1α gene, and protein in the myocardium of NR rats

**Figure 5.**
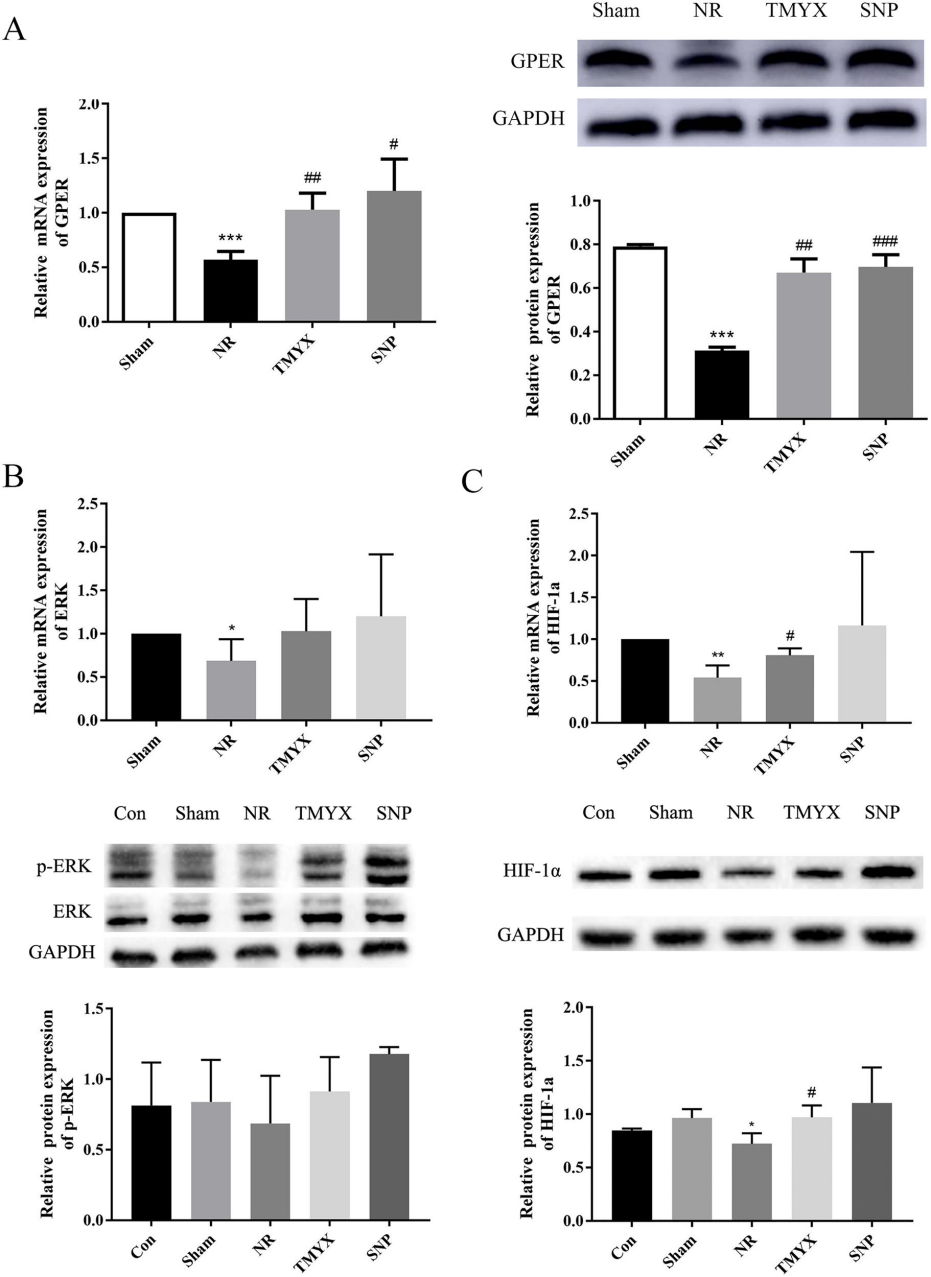
The effects of TMYX on the HIF-1α signaling pathway in NR rats. (A) Effect of TMYX on the expression of the *GPER* gene and protein in the myocardial HIF-1α pathway of NR rats. (B) Effect of TMYX on the expression of the *ERK* gene and p-ERK protein in the myocardial HIF-1α pathway of NR rats. (C) Effect of TMYX on the expression of the *HIF-1α* gene and protein in the myocardial HIF-1α pathway of NR rats. The data are expressed as the mean ± SD; *n* = 3 animals/group; *p < 0.05, **p < 0.01, ***p < 0.001 vs. Sham group; ^#^*p* < 0.05, ^##^*p* < 0.01, ^###^*p* < 0.001 vs. NR group.

The expression of *GPER, ERK, HIF-1α*, GPER, and HIF-1α proteins was significantly decreased in the NR group (*p* < 0.05, *p* < 0.01 and *p* < 0.001), and the expression of the p-ERK protein in the NR group showed a decreasing trend, compared with that in the Sham group (Figure 5(A–C)). TMYX treatment increased the expression levels of *GPER, HIF-1α* gene, and protein (*p* < 0.05, *p* < 0.01), while the expression of *ERK* and p-ERK proteins in the TMYX group showed an increasing trend, compared with that in the NR group. These results indicate that TMYX promotes the expression of the *GPER* gene and protein to regulate HIF-1α signaling.

### Effect of TMYX on diastolic function of isolated coronary microvasculature by the HIF-1α pathway

**Figure 6.**
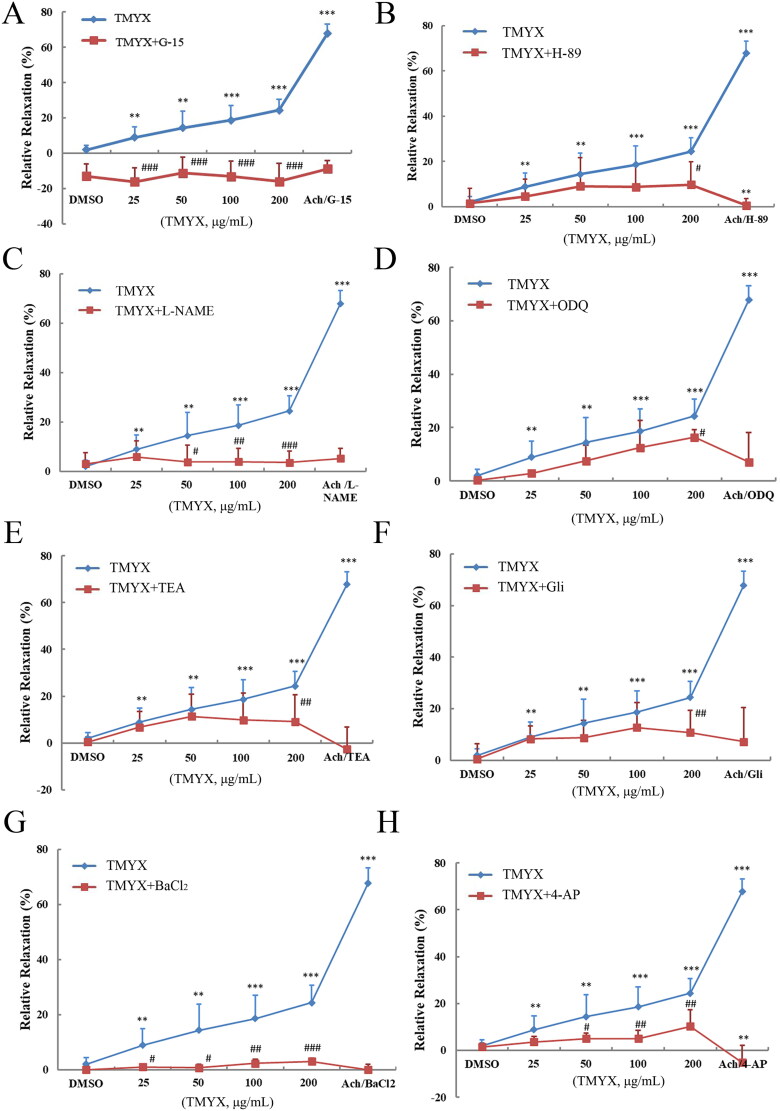
Effect of TMYX on the diastolic function of isolated coronary microvasculature by the HIF-1α pathway. (A) Effect of GPER inhibitor on the diastolic function of isolated coronary microvasculature of TMYX. (B) Effect of PKA inhibitor on the diastolic function of isolated coronary microvasculature of TMYX. (C) Effect of eNOS inhibitor on the diastolic function of isolated coronary microvascular of TMYX. (D) Effect of sGC inhibitor on the diastolic function of isolated coronary microvasculature of TMYX. (E–H) Effect of four K^+^ channel inhibitors on the diastolic function of isolated coronary microvascular of TMYX. The data are expressed as the mean ± SD; *n* = 3 animals/group; **p < 0.01, ***p < 0.001 vs. DMSO group; ^#^*p* < 0.05, ^##^*p* < 0.01, ^###^*p* < 0.001 vs. TMYX group.

The results showed that TMYX enhanced the diastolic function of the coronary microvasculature *in vitro* (Figure 6(A–H)), but this effect was inhibited by G-15, H-89, L-NAME, ODQ, and four K^+^ channel inhibitors (*p* < 0.05, *p* < 0.01, and *p* < 0.001, respectively). This indicates that TMYX can activate GPER to regulate the HIF-1α pathway and downstream potassium channels, and ultimately play a role in the diastolic coronary microvascular function and alleviating NR.

### Effect of TMYX on myeloperoxidase MPO in NR rats

**Figure 7.**
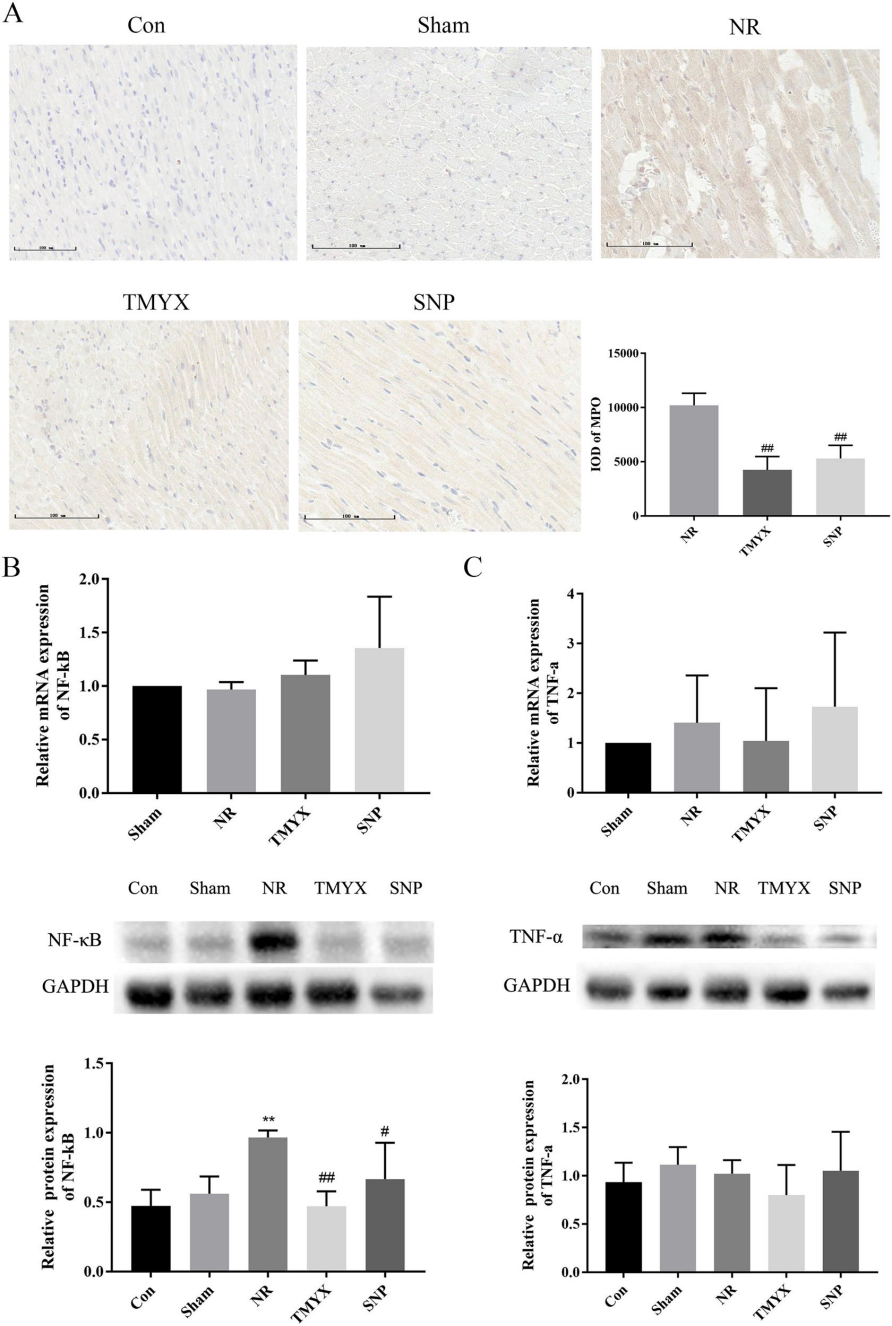
The effects of TMYX on inflammatory signaling pathway in NR rats. (A) Effect of TMYX on myeloperoxidase MPO in NR rats (×200, *n* = 3). (B) Effect of TMYX on the expression of the *NF-κB* gene and protein in NR rats. (C) Effect of TMYX on the expression of the *TNF-α* gene and protein in NR rats. The data are expressed as the mean ± SD; *n* = 3 animals/group; **p < 0.01 vs. Sham group; ^#^*p* < 0.05, ^##^*p* < 0.01 vs. NR group.

Elevated MPO levels in circulation are associated with inflammation and increased oxidative stress. Multiple lines of evidence suggest an association between MPO and cardiovascular disease (CVD) including coronary artery disease, congestive heart failure, myocardial ischemia/reperfusion-related injury. In this regard, MPO may be seen as a mediator or an instrument through which inflammation promotes CVD at the molecular and cellular level (Ndrepepa 2019). As shown in Figure 7(A), MPO was primarily expressed in the cytoplasm of cardiomyocytes in the myocardial layer. Compared with those in the Con group, the sham operation had no effect on MPO expression. MPO expression in the NR group was higher than that in the Sham group. Compared to the NR group, MPO expression decreased in the TMYX and SNP groups (*p* < 0.01), which indicated TMYX could reduce the inflammatory reaction of myocardial tissue.

### Effect of TMYX on the expression of the NF-κB, TNF-α gene and protein in the myocardium of NR rats

NF-κB and TNF-α are the key mediators of inflammation. As shown in Figure 7(B–C), compared with the Sham group, the expression of NF-κB protein (*p* < 0.01) and *TNF-α* gene in the NR group was increased. The expression of NF-κB protein in the TMYX and SNP groups was significantly decreased (*p* < 0.05, *p* < 0.01), the expression of the *TNF-α* gene in the TMYX group is decreased, compared with that in the NR group. However, the expression of the *NF-κB* gene was not significantly different. These results indicate that TMYX decreased the expression of NF-κB protein, but not the gene. The expression of the *TNF-α* gene was not significantly different due to large individual differences. The expression of the TNF-α protein in the TMYX group showed a decreasing trend. These results indicated that TMYX has a trend to decrease the expression of the *TNF-α* gene and protein. Taken together, these data indicated that TMYX could reduce the inflammatory reaction of myocardial tissue.

## Discussion

The NR phenomenon is a complex pathological process that starts in the ischemic phase, deteriorates in the reperfusion phase, and takes myocardial microcirculation disturbances as the pathological core. In humans, NR is caused by a variable combination of four pathogenetic components: distal atherothrombotic embolization, ischemic injury, reperfusion injury, and susceptibility of the coronary microcirculation to injury (Annibali et al. 2022). Current prevention and treatment methods for NR mainly include drug and device therapies (Zhang et al. 2021; Khan et al. 2022). Nevertheless, there are few effective drugs for the clinical treatment of NR.

Our previous research showed that TMYX attenuates myocardial NR after ischemia and reperfusion by activating the PI3K/Akt/eNOS, cAMP/PKA, and NO/cGMP pathways and regulating apoptosis, further upregulating NO activity, and relaxing coronary microvessels. This result suggests that TMYX has the potential to attenuate NR with multiple components, pathways, and targets. Network pharmacology is an emerging discipline based on systems biology, which is used to analyze biological system networks to select specific signal nodes so that the potential interaction between drugs and targets can be accurately determined (Li et al. 2021). This study identified active compounds, potential therapeutic targets, and pathways of TMYX through network pharmacology and explored the mechanism of TMYX on NR.

In this study, 92 active compounds in TMYX with 69 targets were identified, suggesting that TMYX exerts its pharmacological effects in the treatment of NR *via* multiple targets. Quercetin, luteolin, kaempferol, and lysine were identified as the most active compounds, with the top four degrees. As for quercetin, existing studies have indicated that quercetin attenuates myocardial ischemia-reperfusion injury *via* downregulation of the HMGB1-TLR4-NF-κB signaling pathway (Dong et al. 2018). Quercetin improves ischemia/reperfusion-induced cardiomyocyte apoptosis *in vitro* and *in vivo via* SIRT1/PGC-1α signaling (Tang et al. 2019). In addition, quercetin postconditioning attenuates myocardial ischemia/reperfusion injury in rats through the PI3K/Akt pathway (Wang et al. 2013). Luteolin alleviates myocardial ischemia-reperfusion injury in rats *via* the Siti1/NLRP3/NF-κB pathway (Zhao et al. 2020) and activation of peroxiredoxin II (Wei et al. 2018). Luteolin can ameliorate impaired mitochondrial morphology, regulate the MAPK pathway (Yu et al. 2015), and downregulate the TLR4-mediated NF-κB/NLRP3 inflammasome to protect against myocardial ischemia-reperfusion injury (Zhang et al. 2017). Kaempferol can protect against myocardial ischemia/reperfusion injury by activating the PI3K/Akt/GSK-3beta pathway (Wang et al. 2017), antioxidant activity, and inhibition of glycogen synthase kinase-3β (Zhou et al. 2015). With regard to lysine, inhibition of the protein SET domain-containing lysine methyltransferase 7 attenuates hypoxia/reoxygenation-induced injury of cardiomyocytes *via* the downregulation of Keap1 and promotion of the Nrf2-mediated anti-oxidation signaling pathway (Dang et al. 2018). Some data suggest a role for SIRT1-mediated lysine deacetylation in the mechanism of acute ischemic preconditioning; inhibition of SIRT1, either directly or by restricting the availability of its substrate NAD^+^, inhibits ischemic preconditioning (Nadtochiy et al. 2011). Myocardial ischemia-reperfusion can lead to three serious complications: malignant arrhythmia, myocardial stunning, and NR. Overall, TMYX is a multi-component formula with multi-target therapeutic effects.

PPI network analysis demonstrated that the targets linked to NR were NOS3, ALB, CXCL8, IL10, MMP9, IL6, CAT, and TNF; targets (NOS3, TNF) closely related to NR showed a good affinity with the main active ingredient in molecular docking experiments. We found that most hub targets were involved in inflammation, vascular endothelial function, and angiogenesis. The result of KEGG pathway enrichment demonstrated that the anti-NR effect of TMYX is involved in the HIF-1α, cGMP-PKG, NF-κB, and TNF signaling pathways. Preliminary research has shown that TMYX attenuates myocardial NR after ischemia and reperfusion by activating the cGMP-PKG signaling pathway (Chen et al. 2021). This also confirmed the accuracy of the network pharmacology prediction method.

**Figure 8.**
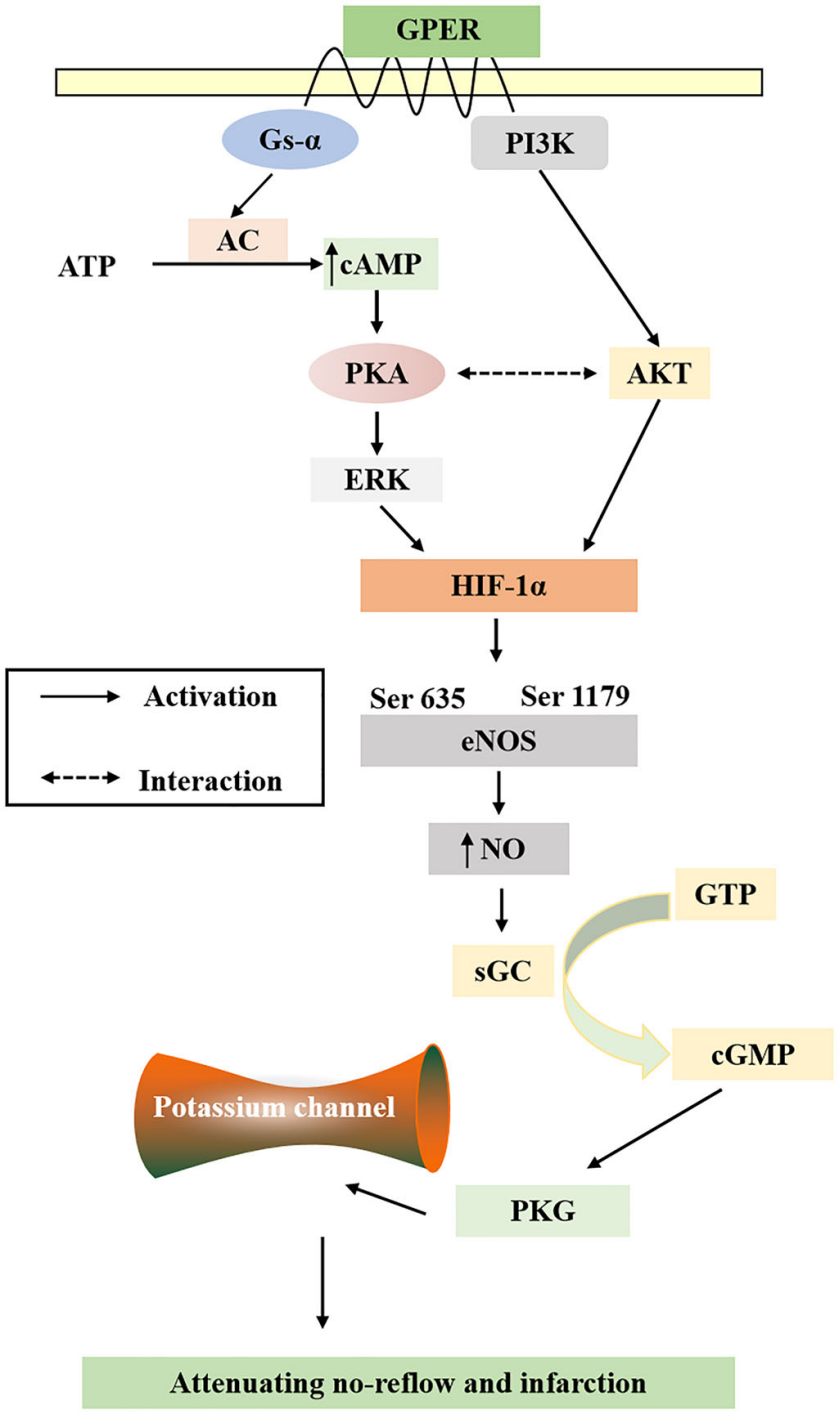
The signaling pathway of TMYX against myocardial no-reflow.

HIF-1*α*, an oxygen-sensitive transcriptional activator that is a major regulator of the hypoxic response to ischemia, plays a pivotal role in angiogenesis which can increase oxygen delivery (Datta Chaudhuri et al. 2021). HIF-1*α* is involved in myocardial remodeling and peri-infarct vascularization in the ischemic heart (Kido et al. 2005). HIF-1*α* activity has been demonstrated to be regulated by the protein kinase AKT and ERK phosphorylation (Lv et al. 2022), and it is noteworthy that AKT and ERK have both been implicated as mediators of cardioprotection (Sumida et al. 2010). Activation of GPER exerts a protective effect in ischemia-reperfusion models and relaxes arteries *in vitro*. GPER-induced relaxation of porcine coronary arteries is mediated by cAMP/PKA signaling (Yu et al. 2014). PKA enhances the *HIF-1α* transcriptional activity and target gene expression in HeLa cells and rat cardiomyocytes (Bullen et al. 2016). Numerous reports have documented that HIF-1*α* directly influences the expression of eNOS (Rajendran et al. 2022), by activating eNOS and producing NO. As the main receptor of NO, sGC catalyzes the conversion of GTP to cGMP to regulate PKG, and PKG regulates K^+^ channels in vascular smooth muscle cells, thereby regulating vascular tension and playing an important role in the process of myocardial ischemia/reperfusion injury and NR (Cohen et al. 2006; Hansen et al. 2021). *In vivo* experiments demonstrated that TMYX promoted the expression of the *HIF-1α* gene and protein. As for p-ERK, TMYX showed an increasing trend, indicating that the HIF-1α pathway is involved in the NR of TMYX. Subsequent experiments also need to include an HIF-1α blocker for verification. *In vitro* experiments showed that TMYX could enhance the diastolic function of coronary microvasculature, but this effect was inhibited by G-15, H-89, L-NAME, ODQ, and four K^+^ channel inhibitors. This indicates that TMYX can activate GPER to regulate the HIF-1α pathway and downstream potassium channel, and ultimately play a role in diastolic coronary microvascular function and alleviating NR (Figure 8).

The inflammatory response, as an essential component of cardiac ischemia/reperfusion (I/R) injury, has been suggested to contribute to myocardial NR. NF-κB and TNF-α are the key mediators of inflammation. Some data suggest that inhibition of NF-κB and TNF-α may reduce I/R-associated myocardial NR by reducing myocardial inflammation (Yu et al. 2021). Our results showed that TMYX reduced neutrophil infiltration and MPO expression, and we used WB and real-time PCR experiments for further verification. The results showed that TMYX decreased the expression of NF-κB protein and tended to decrease the expression of the *TNF-α* gene and protein, indicating that TMYX reduces the effect of the inflammatory response to extenuate NR.

Moreover, some critical targets and active compounds may be ignored because of incomplete database information. Our current experiment and network pharmacology results confirm that TMYX exerts its pharmacological effects in the treatment of NR *via* multiple targets, and provide directions for subsequent research. But the contribution of each pathway was not detected, and the mechanisms should be further investigated.

## Conclusions

We performed network pharmacology and experimental evaluations to reveal the pharmacological mechanism of TMYX against NR. Moreover, a further validation experiment illustrated that TMYX alleviates NR and substantially ameliorates NR by activating GPER to regulate HIF-1α signaling and downstream potassium channels to relax coronary microvasculature and inhibiting the expression of inflammatory factors.

## Data Availability

The datasets used and/or analyzed during the current study are available from the corresponding author on reasonable request.
